# P(*N*-Phenylmaleimide-Alt-Styrene) Introduced with 4-Carboxyl and Its Effect on the Heat Deflection Temperature of Nylon 6

**DOI:** 10.3390/ma11112330

**Published:** 2018-11-20

**Authors:** Yufei Liu, Min He, Daohai Zhang, Qian Zhao, Yang Li, Shuhao Qin, Jie Yu

**Affiliations:** 1 Department of Polymer Material and Engineering, College of Materials and Metallurgy, Guizhou University, Guiyang 550025, China; feiliuyu1990@163.com (Y.L.); zhangdaohai6235@163.com (D.Z.); 15036531454@139.com (Q.Z.); rrzyaj@gmail.com (Y.L.); 2 National Engineering Research Center for Compounding and Modification of Polymeric Materials, Guiyang 550014, China; qinshuhao@126.com

**Keywords:** heat-resistant, nylon 6, P(*N*-phenylmaleimide-alt-styrene), carboxyl, styrene

## Abstract

P(*N*-phenylmaleimide-alt-styrene) (P(NPMI-alt-St)) and P(*N*-(4-carboxyphenyl)maleimide-alt-styrene) (P(CPMI-alt-St)) were designed and synthesized via free radical copolymerization. Fourier transform infrared spectroscopy (FT-IR), nuclear magnetic resonance spectroscopy (^1^H NMR and ^13^C NMR), gel permeation chromatography (GPC), and differential scanning calorimetry (DSC) were used to confirm the structure of P(NPMI-alt-St) and P(CPMI-alt-St). Next, the effect of P(CPMI-alt-St) on the heat deflection temperature (*HDT*) of nylon 6 was studied. In comparison to the PA6/P(NPMI-alt-St) blend, with the addition of 10 wt %, the *HDT* value of the PA6/P(CPMI-alt-St) blend increased by 15.7 °C, and the glass transition temperature (*T*g) by Dynamic mechanical analysis (DMA) increased 2.3 °C. According to the analysis of DMA, dynamic viscosity, and the SEM of PA6 and its blends, P(CPMI-alt-St) promoted its compatibility with PA6, and promoted the storage modulus and dynamic viscosity of the blends. Thus, the introduction of 4-carboxyl can significantly improve the effect of P(CPMI-alt-St) on the heat resistance modification of nylon 6.

## 1. Introduction

Since its discovery as a heat-resistant agent in 1981 by a Japanese catalyst chemical company, *N*-phenylmaleimide (NPMI) and its copolymers have been studied extensively [1,2,3,4] and used widely as a material in home appliances, automobiles, and the electronics industry [5,6,7]. The presence of a five-membered ring of 1,2-vinyl double substitution is a key feature that not only determines the heat resistance, but can also be exploited for further polymerization via free radical copolymerization; examples of comonomers include styrene [8,9,10], maleic anhydride [11], methyl acrylate [12,13], and chloroethylene [14].

Heat deflection temperature (*HDT*) is a widely employed parameter for quality control and material development in industry and can be taken as the material’s ultimate use point. In order to improve the *HDT*, NPMI derivatives and their copolymers have attracted considerable interest with respect to applications in acrylonitrile butadiene styrene (ABS) [15] and poly(vinyl chloride) (PVC) [16,17]. Jianting Dong and his co-workers [11,12,13,14,15] synthesized an *N*-phenylmaleimide (NPMI)-styrene(St)-maleic anhydride (MAH) copolymer (NSM copolymer), and found that the Vicat softening point temperature of the ABS/NSM blend was significantly enhanced by increasing the NSM content. In addition, M. Yuksel et al. [18] found that the glass transition temperature and thermal stabilities of the liquid crystalline moiety containing *N*-cyclohexylmaleimide copolymers were increased by increasing the *N*-substituted maleimide (*N*-cyclohexylmaleimide) content. Last, but not least, Shantilal Oswal et al. [19] synthesized an *N*-(4-carboxyphenyl)maleimide (CPMI)/methyl methacrylate (MMA) copolymer and a CPMI homopolymer, and found that CPMI enhanced the initial decomposition temperature of the copolymer, because the incorporated five-membered planar cyclic structure in the copolymer chain enhanced the thermal stability of the copolymer. The above studies show that NPMI derivatives and their copolymers play a positive role in improving the heat resistance of polymers such as ABS and PVC. However, the heat resistance modification of aliphatic series nylon via NPMI derivatives and their copolymers have not been reported to our knowledge.

Nylon 6 (PA6) is a kind of semi-crystalline polyamide material [20,21] with an extensive application range in the aerospace, electrical equipment, and automobile industries due to its properties, i.e., high toughness and high-performance. However, PA6 has a relatively low heat resistance when compared with other high-performance engineering plastics, such as polycarbonate (PC), polyphenyl ether (PPO), and polyphenylene sulfide (PPS), which limits PA6’s use in high-heat resistant applications, such as automotive engines, fuel systems, and electrical equipment. Enlightened by the above publications, we designed and synthesized an *N*-(4-carboxyphenyl)maleimide and its copolymer. The aim of the paper was to study the P(*N*-phenylmaleimide-alt-styrene) introduced with 4-carboxyl and its effect on the *HDT* of a blend of nylon 6 and P(*N*-(4-carboxyphenyl)maleimide-alt-styrene).

## 2. Materials and Methods

### 2.1. Materials

Maleic anhydride (MAH) was purchased from the Tianjin Damao Chemical Reagent Factory (Tianjin, China) and used as received. Hydroquinone was purchased from Tianjin Commie Chemical Reagent Co. Ltd. (Tianjin, China) and used as received. Styrene (St, CR) was purchased from Sinopharm Chemical Reagent Co. Ltd. (Shanghai, China), and was passed over an aluminum oxide column before usage to remove any inhibitors. Para aminobenzoic acid was purchased from Shanghai Aladdin Biochemical Technology Co. Ltd. (Shanghai, China) and used as received. Benzoyl peroxide was recrystallized from methyl alcohol and stored in the fridge. Aniline was purchased from Chengdu Jinshan Chemical Reagent Co. Ltd. (Chengdu, China) and used as received. Acetic anhydride and acetone were purchased from Chongqing Sichuan East Chemical (Group) Co. Ltd. (Chongqing, China) and used as received. *N*,*N*-dimethyl formamide was purchased from Taicang Hushi Test Reagent Co. Ltd. (Taicang, China) and used as received. Cyclohexanone was purchased from the Chengdu Kelong Chemical Reagent Plant (Chengdu, China) and used as received. Sodium acetate anhydrous and thionyl chloride were also purchased from the Chengdu Kelong Chemical Reagent Plant and used as received. Nylon 6 (PA6, YH-800) was purchased from Yueyang Petrochemical Co. Ltd. (Yueyang, China) and used as received.

### 2.2. Synthesis of N-Phenylmaleimide (IUPAC Name: 1-phenyl-1H-pyrrole-2,5-dione; NPMI)

**Scheme 1:**
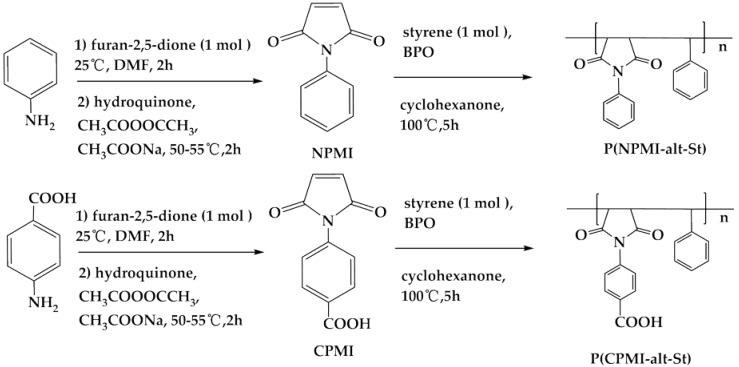
The general synthetic of P(NPMI-alt-St) and P(CPMI-alt-St). BPO, benzoyl peroxide.

As shown in Scheme 1, the solution of aniline (93.06 g, 1 mol) in *N*,*N*-dimethyl formamide (DMF, 100 mL) was gradually added over a period of 15 min to a well-stirred solution of maleic anhydride (98.1 g, 1 mol) in DMF (500 mL). The solution was stirred for 2 h at room temperature. Then, dusty sodium acetate (12.0 g), hydroquinone (10 g), and acetic anhydride (50 mL) was added to the resulting clear solution and heated up to 50 °C in an oil bath for 2 h. A light yellow mass of *N*-phenylmaleimide (NPMI, Figure S1: ^1^H and ^13^C NMR spectra of NPMI in CDCl_3_), which was obtained by adding the solution to crushed ice, was washed several times with water and then dried in an air oven at 50–60 °C.

### 2.3. Synthesis of N-(4-Carboxyphenyl)Maleimide (IUPAC Name: 4-(2,5-Dioxo-2,5-Dihydro-1H-pyrrol-1-yl)benzoic Acid; CPMI)

As shown in Scheme 1, the solution of para aminobenzoic acid (137.05 g, 1 mol) in *N*,*N*-dimethyl formamide (DMF, 100 mL) was gradually added over a period of 15 min to a well-stirred solution of maleic anhydride (98.1 g, 1 mol) in DMF (500 mL). The solution was stirred for 2 h at room temperature. Then, dusty sodium acetate (12.0 g), hydroquinone (10 g), and acetic anhydride (50 mL) was added to the resulting clear solution and heated up to 50 °C in an oil bath for 2 h. A light yellow mass of *N*-(4-carboxyphenyl)maleimide (CPMI, Figure S2: ^1^H and ^13^C NMR spectra of CPMI in DMSO), obtained by adding the solution to crushed ice, was washed several times with water and then dried in an air oven at 60–70 °C.

### 2.4. Free Radical Copolymerization of NPMI and St

As shown in Scheme 1, the free radical copolymerization of P(NPMI-alt-St) was carried out in a 5 L mouth flask purged with dry nitrogen. The polymerization process was as follows: NPMI (217 g) and St (104 g) were added and dissolved in cyclohexanone (3 L) by stirring at 76 °C for 40 min. Then, the initiator, BPO (7.5 g), was added after the system was flushed with nitrogen to remove the oxygen. The reactor was heated by external circulating heated silicon oil, and the copolymerization was carried out at 100 °C for 4 h under a nitrogen atmosphere and then cooled down to room temperature. The copolymer was then dissolved in acetone, and the solution was poured into an excess of methanol to remove residual monomers and initiators. This procedure was repeated twice, and the final product was dried under vacuum at 130 °C up to constant weight.

### 2.5. Free Radical Copolymerization of CPMI and St

As shown in Scheme 1, the free radical copolymerization of P(CPMI-alt-St) was the same as that of P(NPMI-alt-St). However, after being dissolved in acetone, it needed to be poured into an excess of petroleum ether and methanol to remove the residual monomers and initiators.

### 2.6. Specimen Preparation of PA6 and Its Blends

After being dried under vacuum for 6 h at 80 °C, PA6 and its blends were prepared by mixing PA6 and P(NPMI-alt-St) (or P(CPMI-alt-St)) (*w*PA6:*w*P(NPMI-alt-St) (P(CPMI-alt-St)) = 90:10) in a co-rotating twin-screw extruder (TSE–40A, L/D = 40, D = 40 mm, Coperion Keya Machinery Co. Ltd., Nanjing, China) at 200–240 °C with a speed of 240 r/s. PA6 and its blends were dried for 24 h at 80 °C to remove water. Then, PA6 and its blends were injection-molded (type CJ80M3V, Chen De Plastics Machinery Co. Ltd., Chengde, China) at 240 °C into various specimens for testing and characterization.

### 2.7. General Characterization Methods

The 400 MHz ^1^H and 101 MHz ^13^C NMR spectra were recorded on a Bruker Ascend400 spectrometer (Billerica, MA, USA). The ^1^H and ^13^C NMR spectra were referenced internally to the solvent peaks.

FT-IR spectra from 4000 to 400 cm^−1^ were recorded on a Nicolet 6700 spectrometer (KBr discs, Thermo Fisher Scientific, Waltham, MA, USA). The scanning rate was 32 min^−1^, and the differentiate rate was 4 cm^−1^.

GPC analyses were performed in THF (1.0 mL/min, 30 °C) using a Viscotek TDA302 (Malvern Panalytical Ltd., Malvern, UK) with a WL. M GPC solvent/sample module. Polystyrene standards (M_w_ = 105 k) were used for calibration. The absolute molecular mass was measured by a viscosity detector, a concentration detector, and a scattering detector.

Differential scanning calorimetry (DSC) measurements were performed on a TA Q20 system (TA Instrument, New Castle, DE, USA) at a scan rate of 10 °C/min between 40 and 270 °C and protected by nitrogen. The first heating cycle from 40 to 270 °C was used to eliminate the thermal history; keeping 270 °C for three minutes and then it went down to 40 degrees at a rate of 10 °C/min. The results reported were from the second heating cycle. The percent crystallinity was determined by dividing the heat of fusion value by the heat of fusion of 100% crystalline PA6 per the equation:

(1) $$X_{c}=\frac{∆H_{m}}{\left(1-x\right)∆H_{m}^{0}}$$

where ∆*H*_m_ is crystallization enthalpy of the samples (J/g), $∆H_{m}^{0}$ is the enthalpy value of the melting of a 100% crystalline form of PA6, which is 230 J/g, and x is the weight fraction of the P(NPMI-alt-St) (or P(CPMI-alt-St)).

Heat deflection temperature (*HDT*) measurements were performed on a Thermal Deformation Temperature Tester (MTS ZWK1000, Shanghai, China). The measurements were carried out according to GB/T1634.1-2004, with a pressure of 0.45 MPa and a temperature rate of 120 °C h^−1^. Four measurements were used for calculating the standard deviation.

Dynamic mechanical analysis (DMA) measurements were performed on a Q800 DMA (TA Instruments, USA). The measurements were carried out at 1 Hz under a heating rate of 2 °C min^−1^. The temperature range was from 40 °C to 140 °C, and the high-temperature measurements were carried out in a stream of dry N_2_.

Dynamic viscosity was performed on an advanced rheometric expansion system (ARES, TA Instrument, New Castle, DE, USA) using the parallel plates mode. The measurements were conducted at 230 °C. The samples were loaded onto the bottom plate of the instrument and allowed to melt. Frequency sweeps at 230 °C were performed in the frequency range of 0.01 rad/s–500 rad/s in the linearity region. A strain of 1% was used, which was in the linear viscoelastic regime for all samples.

Scanning electron microscopy (SEM) images were obtained on a KYKY-2800B (Beijing Branch of the Bureau of Technology Development Co. Ltd. (Beijing, China)) to investigate the impact fracture of the samples, which were etched with tetrahydrofuran for 24 h. SEM graphs of the blends were recorded after gold coating surface treatment with an accelerating voltage of 10 kV.

## 3. Results and Discussion

### 3.1. Characterization of the NPMI and P(NPMI-alt-St)

**Figure 1:**
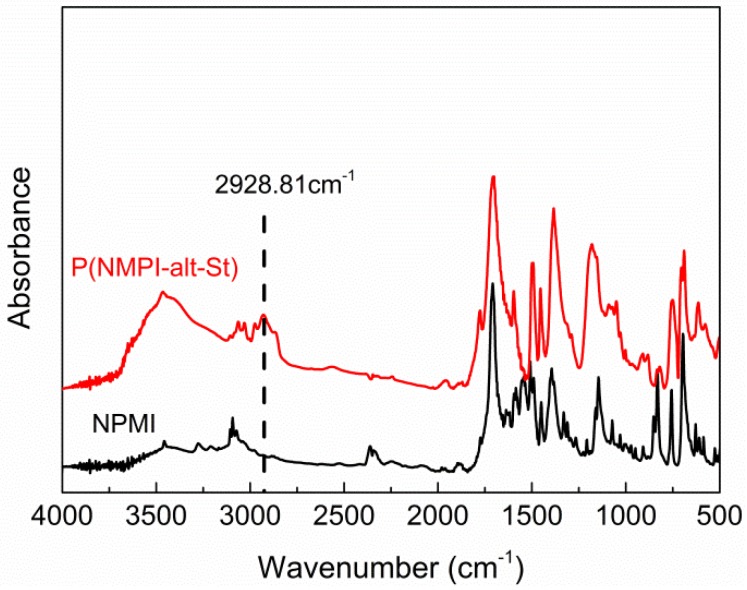
FT-IR spectra of the synthesized NPMI and P(NPMI-alt-St).

**Figure 2:**
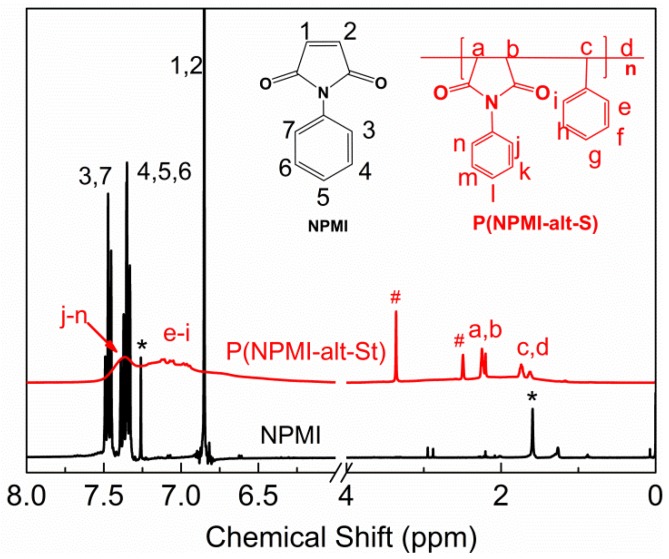
^1^H NMR spectra of the synthesized NPMI and P(NPMI-alt-St). (*) belongs to CDCl_3_ and (#) belongs to DMSO.

**Figure 3:**
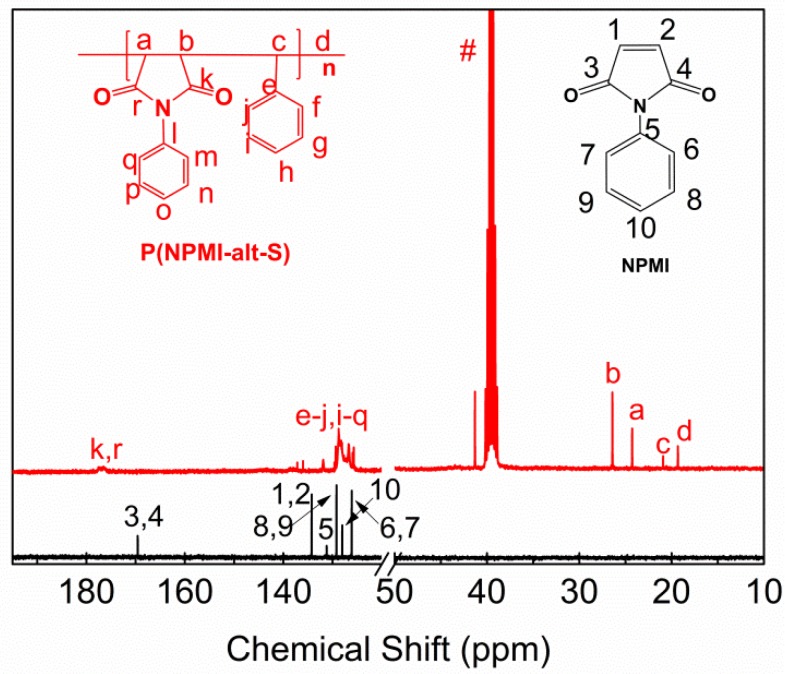
^13^C NMR spectra of the synthesized NPMI and P(NPMI-alt-St). (#) belongs to DMSO.

In the FT-IR spectra, the absorbance band at 2928.81 cm**^−^**^1^ corresponded to the bending vibration of the carbon chain polymer (Figure 1) [22]. This indicated that the polymerization had been carried out. In the ^1^H NMR spectra, the signal at 6.88 ppm was assigned to the protons of HC=CH of NPMI. After the copolymerization, the signal at 6.88 ppm disappeared and signals at 2.18–2.17 appeared (Figure 2); the signals at 2.18–2.17 ppm were assigned to the protons of HC-CH of NPMI in the main chain. Moreover, in the ^13^C NMR spectra, the signal at 134.23 ppm was assigned to the carbons of HC=CH of NPMI, the signal at 134.23 ppm disappeared, and signals at 24.18 and 26.47 appeared (Figure 3).

As reported by Ha [23], triad sequences of styrene (S) and NPMI in a copolymer can be determined by ^13^C NMR. A perfectly alternating sequence has a resonance at 137–140 ppm and a resonance at 33–37 ppm. A polymer that has more styrene (sequences such as SSN to SSS) will experience an upfield shift of these resonances to 145–148 ppm. However, P(NPMI-alt-St) did not have an upfield shift. The disappearance of the vinyl group signals in the ^1^H and ^13^C NMR spectra and the observation of broadened peaks at similar chemical shifts to those of the monomer indicated the successful copolymerization.

According to the GPC analysis in THF, relative to the PS standards, the molecular weight of P(NPMI-alt-St) was *M*_n_ = 12 K g mol**^−^**^1^ (PDI = *M*_w_/*M*_n_ = 1.50) (Figure S3). As a thermoplastic heat-resistant polymer, the glass transition temperature (T_g_) is an important parameter, which is a physical quantity that represents the ability of the molecules to move. The *T*_g_ of PNS (*M*_n_ = 12K g mol**^−^**^1^) was determined by a DSC analysis to be 195.7 °C (Figure S4).

### 3.2. Characterization of the CPMI and P(CPMI-alt-St)

**Figure 4:**
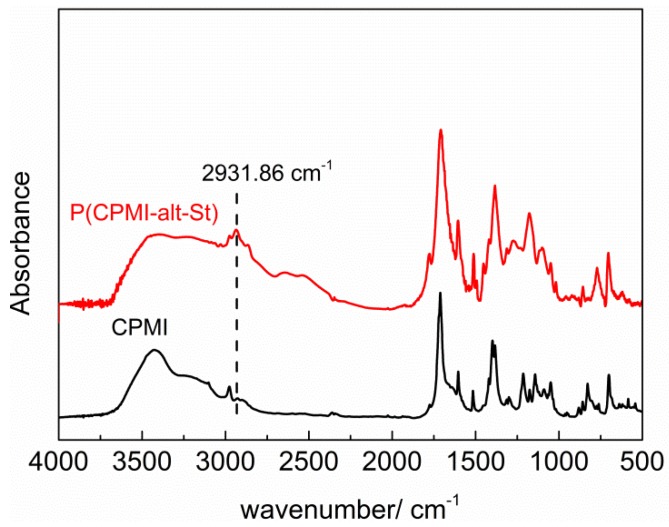
FT-IR spectra of the synthesized CPMI and P(CPMI-alt-St).

**Figure 5:**
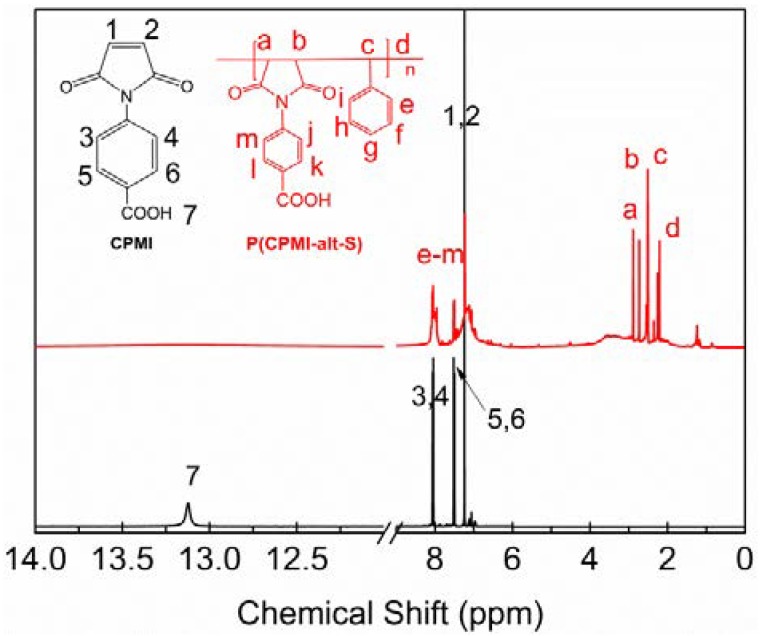
^1^H NMR spectra of the synthesized CPMI and P(CPMI-alt-St).

**Figure 6:**
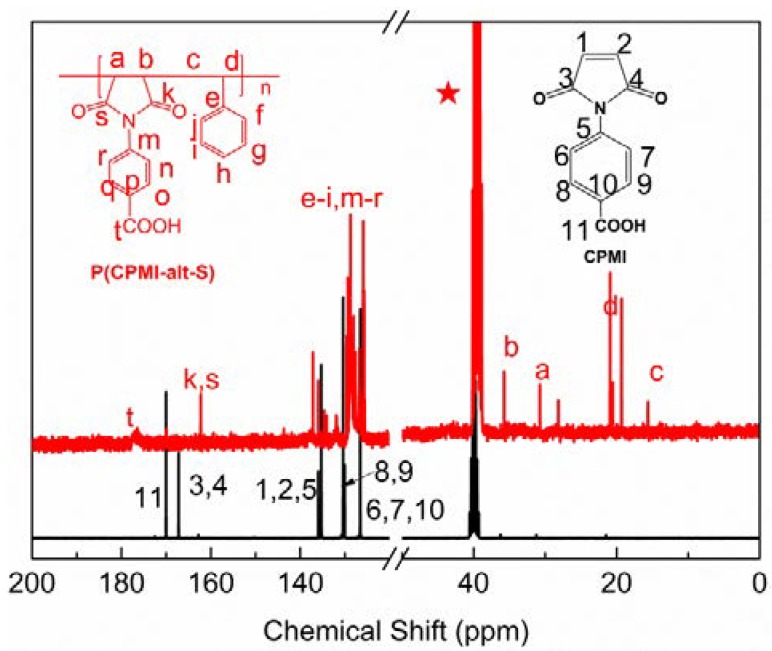
^13^C NMR spectra of CPMI and P(CPMI-alt-St). (★) belongs to DMSO.

In the FT-IR spectra, the absorbance band at 2931.86 cm*^−^*^1^ corresponded to the bending vibration of the carbon chain polymer (Figure 4) [22]. This indicated that the polymerization was carried out. In the ^1^H NMR spectra, the signal at 7.23 ppm was assigned to the protons of HC=CH of CPMI. After the copolymerization, the signal at 7.23 ppm disappeared and signals at 2.71–2.88 appeared (Figure 5); the signals at 2.71–2.88 ppm were assigned to the protons of HC-CH of CPMI in the main chain. Moreover, in the ^13^C NMR spectra, the signal at 135.28 ppm was assigned to the carbons of HC=CH of CPMI, the signal at 135.28 ppm disappeared, and signals at 31 and 35.7 appeared (Figure 6).

According to the GPC analysis in THF, relative to the PS standards, the molecular weight of P(NPMI-alt-St) was *M*_n_ = 9K g mol**^−^**^1^ (PDI = *M*_w_/*M*_n_ = 1.50) (Figure S5). The *T*_g_ of P(CPMI-alt-St) (*M*_n_ = 9k g mol**^−^**^1^) was determined by a DSC analysis to be 208.9 °C (Figure S6). With the same molecular weight, the introduction of the 4-carboxyl of P(CPMI-alt-St) increased the *T*_g_ of the copolymer by 12.3 °C. A single T_g_ further verified that the reaction was an alternating copolymerization rather than a homogenous mixture.

### 3.3. Effect of P(NPMI-alt-St) Induced with 4-Carboxyl on the Nylon 6 Heat-Resistant Modification

**Figure 7:**
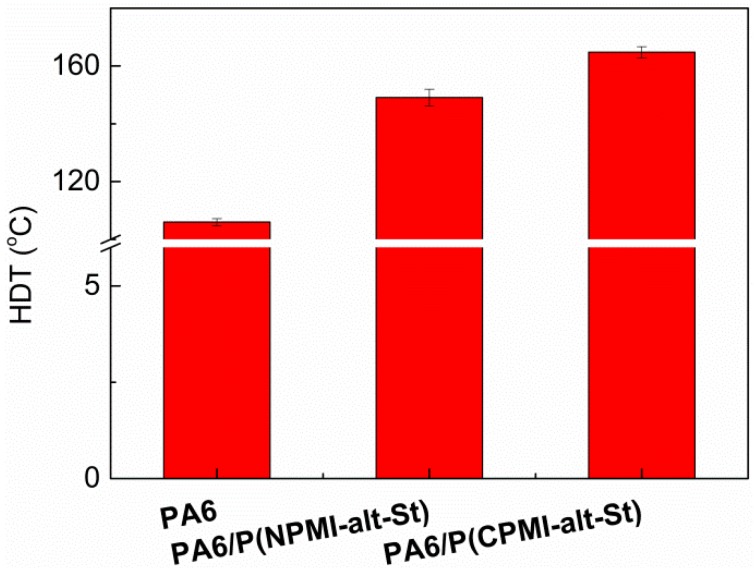
The heat deflection temperature of PA6 and its blends.

Heat deflection temperature (*HDT*) is a widely employed parameter for quality control and material development in industry and can be taken as the material’s ultimate use point [11]. As shown in Figure 7, the *HDT* of virgin PA6 was only 106 °C; this restricts its application in automobiles and domestic appliances. However, the *HDT* value was increased by more than 43.1 °C with the addition of 10 wt % P(NPMI-alt-St), and more than 58.8 °C with the addition of 10 wt % P(CPMI-alt-St). In stark contrast, when compared to the PA6/P(NPMI-alt-St) blend, the *HDT* value of the PA6/P(CPMI-alt-St) blend increased by 15.7 °C. This shows that 4-carboxyl played a positive role in increasing the *HDT* of nylon 6 by P(CPMI-alt-St). In order to study the effort mechanism of the P(CPMI-alt-St) on the *HDT* of PA6, differential scanning calorimetry (DSC), dynamic thermal mechanical analysis (DMA), dynamic viscosity (η), and scanning electron microscopy (SEM) were carried out.

### 3.4. DSC Analysis

**Figure 8:**
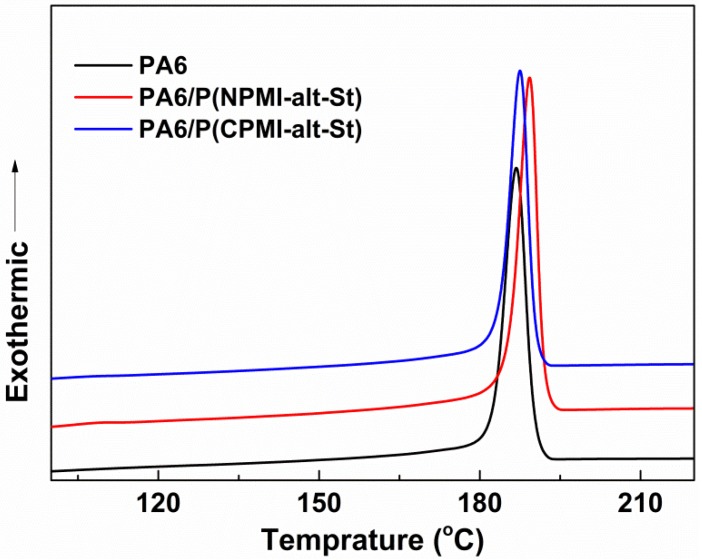
Crystallization (cooling) curves of PA6 and its blends.

**Figure 9:**
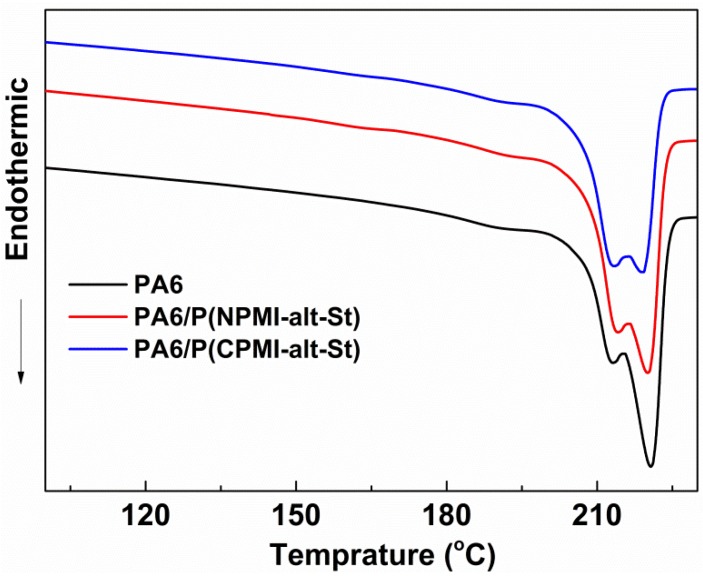
Melting (heating) curves of PA6 and its blends.

From Figure 8 and Table 1, it can be seen that the crystallization peak temperature (*T*_c_) values of the PA6, the PA6/P(NPMI-alt-St) blend, and the PA6/P(CPMI-alt-St) blend were 186.8, 189.3, and 187.5 °C whereas their relative crystallinity (*X*_c_) was 22.8%, 26.3%, and 26.6%, respectively. It was observed that P(NPMI-alt-St) and P(CPMI-alt-St) could slightly enhance the crystallization of PA6, which was demonstrated by *X*_c_. As is widely known, crystallization can improve the heat resistance of polymers [24]. Therefore, P(NPMI-alt-St) and P(CPMI-alt-St), as heterogeneous nucleating agents, promoted nylon 6 crystals, which is one of the reasons why P(NPMI-alt-St) and P(CPMI-alt-St) can be a heat-resistant modification of nylon 6. From Figure 9 and Table 1, the two peaks represented different crystal types: the low-temperature peak represented the melting peak of the γ crystal type, and the high-temperature peak represented the melting peak of the α crystal type. The P(CPMI-alt-St) could induce the transformation of the α crystal type to the γ crystal type because the 4-carboxyl (-COOH) formed a hydrogen bond with the amino (-NH_2_) in nylon 6, inducing nylon to form a γ crystal. Mateusz Z. Brela [25] studied the hydrogen bond dynamics in nylon 6 crystals using IR spectroscopy and molecular dynamics, focusing on the differences between α and γ crystal forms, and found that hydrogen bonds were dynamically favored in the γ form of nylon 6.

### 3.5. DMA Analysis

**Figure 10:**
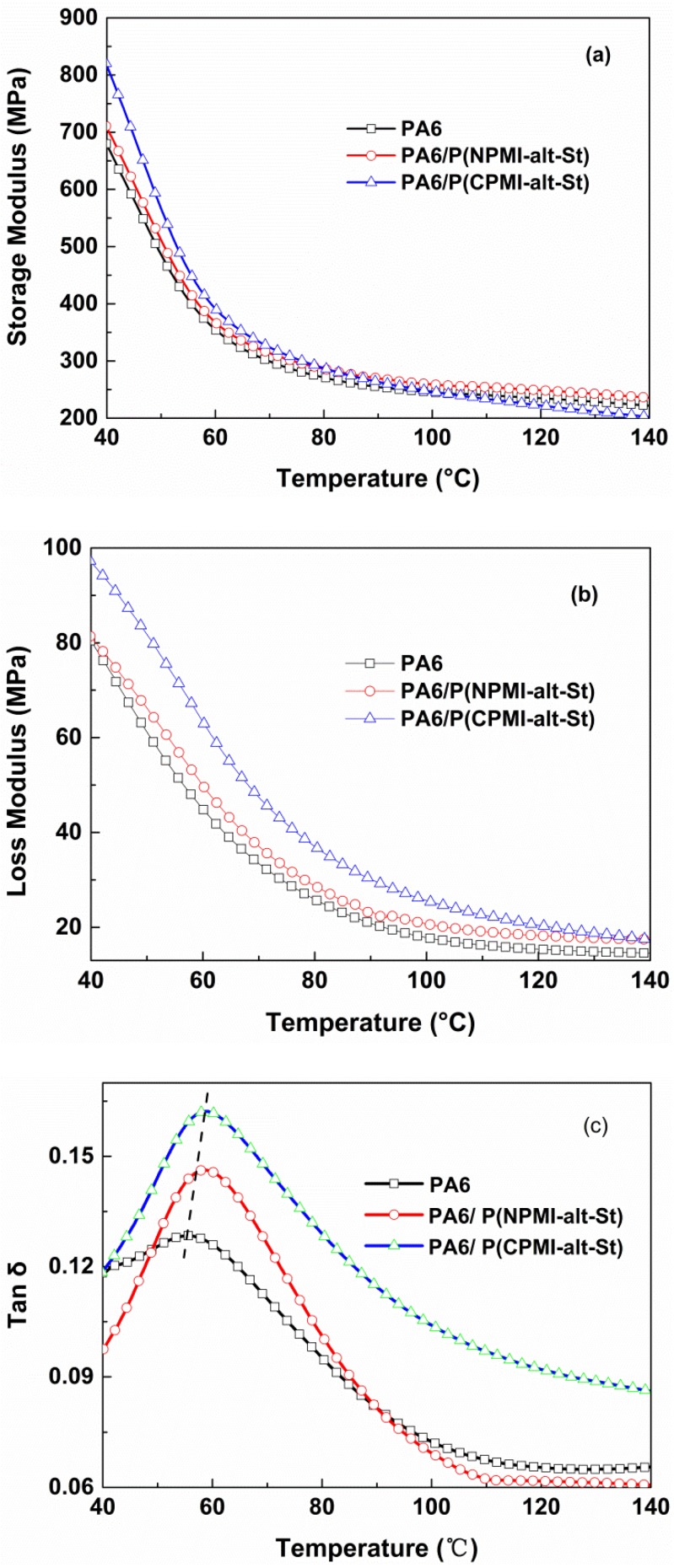
DMA traces of the storage modulus (**a**), loss modulus (**b**), and loss factor (**c**) of PA6 and its blends.

Dynamic thermal mechanical analysis (DMA) data for blends can provide information on the glass transition temperatures of the blends to better understand the phase structure and interphone mixing of the blends [26,27]. The DMA properties of PA6 and its blends are shown in Figure 10. Figure 10a shows that the storage modulus curve changed with an increase in temperature. When P(NPMI-alt-St) and P(CPMI-alt-St) were added, the storage modulus increased when compared with the pure PA6 between 40 and 80 °C, while the storage modulus remained basically constant from 80 to 140 °C. Figure 10b shows that the loss modulus curve changed with a rise in temperature. When P(NPMI-alt-St) and P(CPMI-alt-St) were added, the loss modulus increased significantly when compared with the pure PA6. This indicated that P(NPMI-alt-St) and P(CPMI-alt-St) hindered the movement of PA6 molecules, and improved the intermolecular forces of the PA6 blends [28]. Thus, the introduction of 4-carboxyl made it more effective. Figure 10c shows the change in loss factor (tan δ) with temperature rise. It can be seen from the figure that the glass transition temperature (*T*_g_) of the pure PA6, the PA6/P(NPMI-alt-St) blend, and the PA6/P(CPMI-alt-St) blend was 55.5 °C, 57.2 °C, and 59.5 °C, respectively. Compared to the PA6/P(NPMI-alt-St) blend, the *T*_g_ value of the PA6/P(CPMI-alt-St) blend increased by 2.3 °C. The maximum tan δ of the pure PA6, the PA6/P(NPMI-alt-St) blend, and the PA6/P(CPMI-alt-St) blend was 0.127, 0.146, and 0.162, respectively. The introduction of 4-carboxyl made the tan δ of the PA6/P(CPMI-alt-St) blend increase more than that of the PA6/P(NPMI-alt-St) blend, indicating that P(CPMI-alt-St) made the friction between the molecules bigger and the effect of blocking the movement of molecules was more obvious, which was consistent with the change in *HDT*.

### 3.6. Dynamic Viscosity Analysis

**Figure 11:**
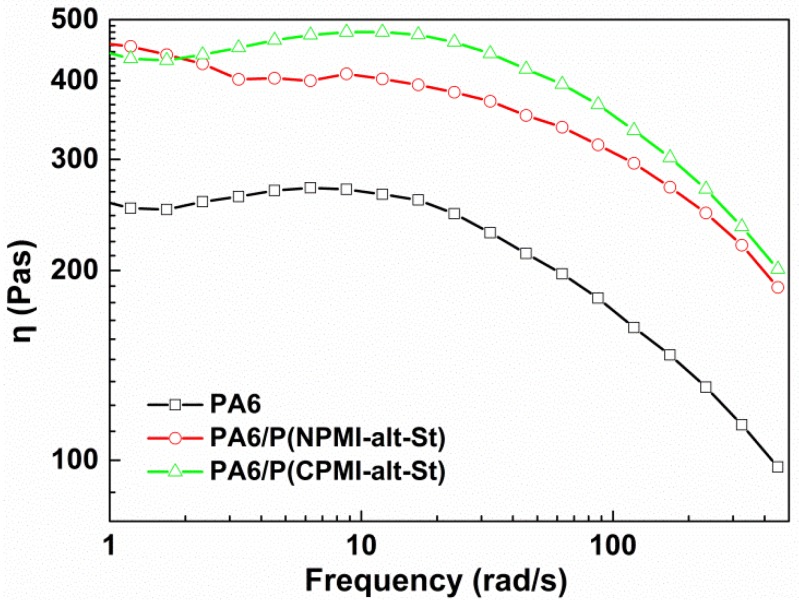
Dynamic viscosity analysis traces of PA6 and its blends.

Dynamic viscosity, which is related to the loss modulus, represents the contribution of viscosity and is the energy dissipation part of the complex viscosity. Figure 11 shows the dynamic viscosity (η) of PA6 and its blends with different functional groups. It can be seen that the η decreased with the increase of frequency, so its rheological behavior was like non-Newtonian fluid [27]. Additionally, the η of the PA6 blends was improved by the addition of P(NPMI-alt-St) and P(CPMI-alt-St). In particular, the η value of PA6/P(CPMI-alt-St) was more than that of P(NPMI-alt-St), which indicated that P(CPMI-alt-St) made the η of the PA6 blends larger, which was consistent with the change in loss modulus and *HDT*.

### 3.7. SEM Analysis

**Figure 12:**
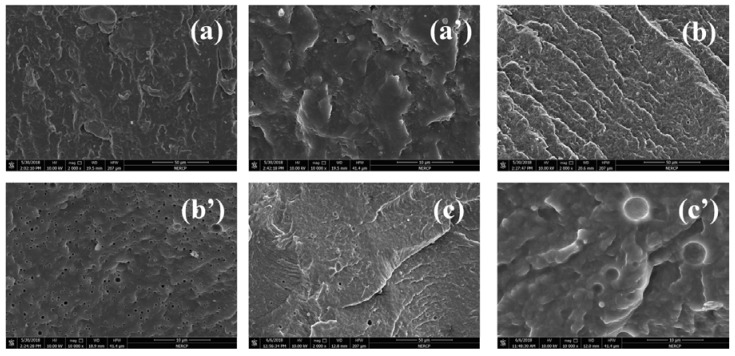
SEM (5000× and 20,000×) of PA6 and its blends (**a**,**a′**) PA6; (**b**,**b′**) PA6/P(NPMI-alt-St) blend; and (**c**,**c′**) PA6/P(CPMI-alt-St) blend.

The morphology of the etched cut surface of the PA6 and its blends is shown in Figure 12. THF was used to dissolve the P(NPMI-alt-St) and P(CPMI-alt-St) fraction of the blend, revealing the PA6’s structure. In the blends, P(NPMI-alt-St) and P(CPMI-alt-St) forms spherical domains uniformly dispersed in the PA6 matrix. Among the PA6/P(NPMI-alt-St) blends, the etched P(NPMI-alt-St) was greater, and the interface was clear, while in the PA6/P(CPMI-alt-St) blend, the etched P(CPMI-alt-St) was lower and the interface was vague. These showed that the introduction of 4-carboxyl made P(CPMI-alt-St) and PA6 more compatible [29]. Thiruvengadam Vedamurthy [30] studied the adhesion of PMMA and PA6 by TEM and found that PMMA and p-Cresol resin-nylon 6 were interconnected by hydrogen bonding to form PMMA-modified p-Cresol resin-nylon 6. The conclusions of this paper are consistent with theirs.

## 4. Conclusions

In conclusion, P(*N*-phenylmaleimide-alt-styrene) (P(NPMI-alt-St)) and P(*N*-(4-carboxyphenyl)maleimide-alt-styrene) (P(CPMI-alt-St)) via free radical copolymerization were successfully designed and synthesized, and the introduction of 4-carboxyl onto P(*N*-phenylmaleimide-alt-styrene) and its effect on the heat deflection temperature (*HDT*) of nylon 6 were studied. The *HDT* values of the PA6/P(NPMI-alt-St) and PA6/P(CPMI-alt-St) blends immensely exceeded that of PA6, while the *HDT* value of the PA6/P(CPMI-alt-St) blend exceeded that of the/P(NPMI-alt-St) blend. The DSC analysis proved that the introduction of 4-carboxyl onto P(NPMI-alt-St) induced the transformation of the α crystal type to the γ crystal type and then promoted the crystallinity of PA6. The DMA and dynamic viscosity analysis proved that the introduction of 4-carboxyl onto P(NPMI-alt-St) enhanced the friction between P(CPMI-alt-St) and PA6. Thus, we anticipate the development of polarity-substituted heat-resistant agents to greatly expand the diversity and application of a range of PA6 materials.

## Figures and Tables

**Table 1:** DSC results of PA6 and its blends.

Samples	*X*_n_ (%)	*T*_c_ (°C)	*T*_m1_ (°C)	*T*_m2_ (°C)
PA6	22.8	186.8	213.2	220.7
PA6/P(NPMI-alt-St)	26.3	189.3	214.6	220.2
PA6/P(NPMI-alt-St)	26.6	187.5	213.4	219.2

## Supplementary Materials

The following are available online at http://www.mdpi.com/1996-1944/11/11/2330/s1, Figure S1: ^1^H and ^13^C NMR spectra of NPMI in CDCl_3_, Figure S2: ^1^H and ^13^C NMR spectra of CPMI in DMSO, Figure S3 GPC trace of P(NPMI-alt-St), Figure S4: DSC trace of P(NPMI-alt-St), Figure S5: GPC trace of P(CPMI-alt-St), Figure S6: DSC trace of P(CPMI-alt-St).
