# Supplementary material for: P(N-Phenylmaleimide-Alt-Styrene) Introduced with 4-Carboxyl and Its Effect on the Heat Deflection Temperature of Nylon 6
Source: Materials (Basel). 2018 Nov 20;11(11):2330. doi: 10.3390/ma11112330 (PMC6267326; doi:10.3390/ma11112330)
Supplement: Supplementary file 1 [file materials-11-02330-s001.pdf]

*materials*

MDPI

*Article*

# P(*N*-Phenylmaleimide-Alt-Styrene) Introducedwith 4-Carboxyl and Its Effect on the Heat Deflection Temperature of Nylon 6

**Yufei Liu [1,2], Min He [1,2,*], Daohai Zhang [1,2], Qian Zhao [1], Yang Li [1], Shuhao Qin [2] and Jie Yu [2,*]**

[1] Department of Polymer Material and Engineering, College of Materials and Metallurgy, Guizhou University, Guiyang 550025, China; feiliuyu1990@163.com (Y.F.L.); zhangdaohai6235@163.com (D.Z.); 15036531454@139.com (Q.Z.); rrzyaj@gmail.com (Y.L.);

[2] National Engineering Research Center for Compounding and Modification of Polymeric Materials, Guiyang 550014, China; qinshuhao@126.com

* Correspondence: hemin851@163.com (M.H.); yujiegz@126.com (J.Y.)

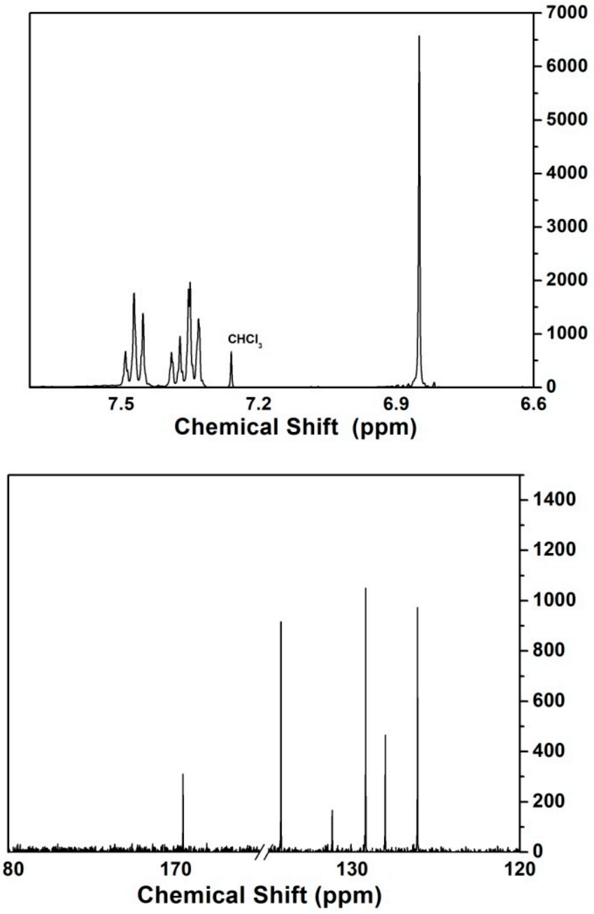


**Figure S1.** $^1$H and $^{13}$C NMR spectra of NPMI in $CDCl_3$.

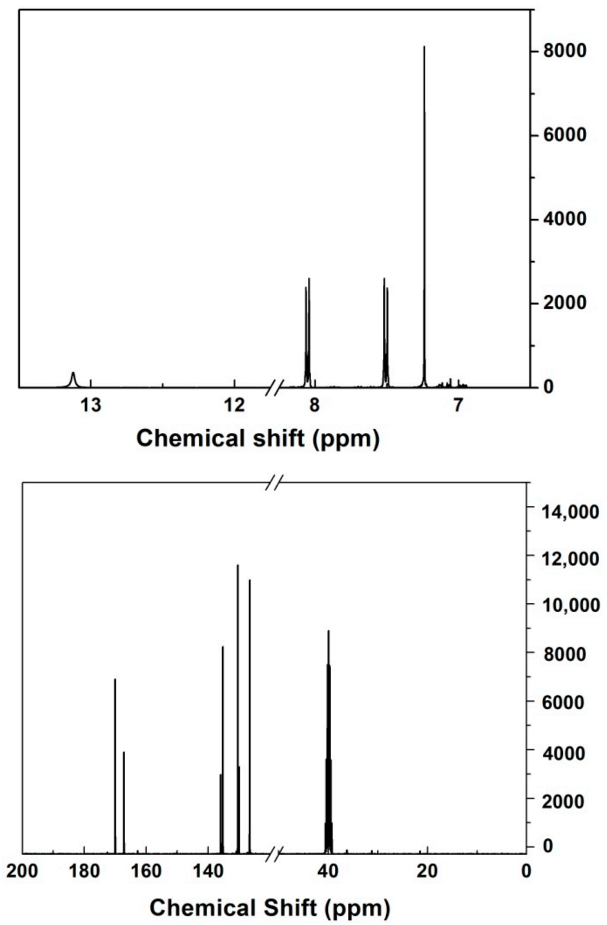


**Figure S2.** $^1H$ and $^{13}C$ NMR spectra of CPMI in DMSO.

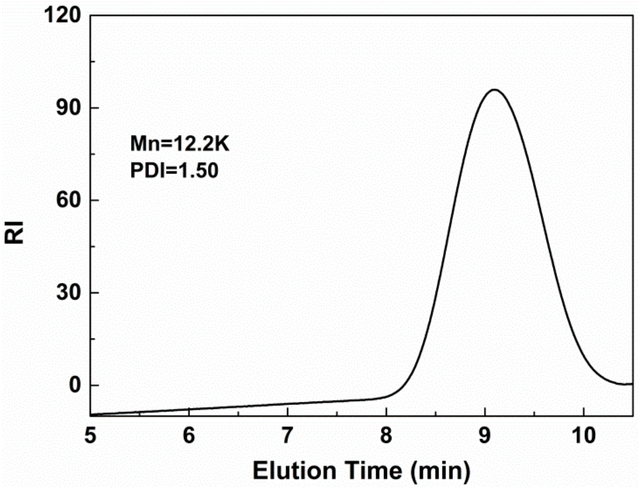


**Figure S3.** GPC trace of P(NPMI-alt-St).

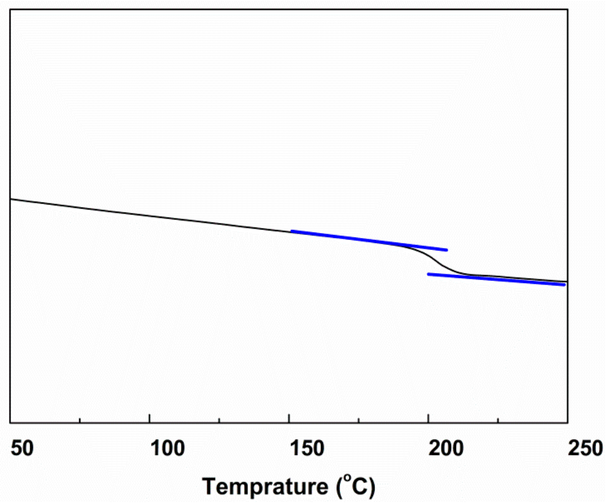


**Figure S4.** DSC trace of P(NPMI-alt-St).

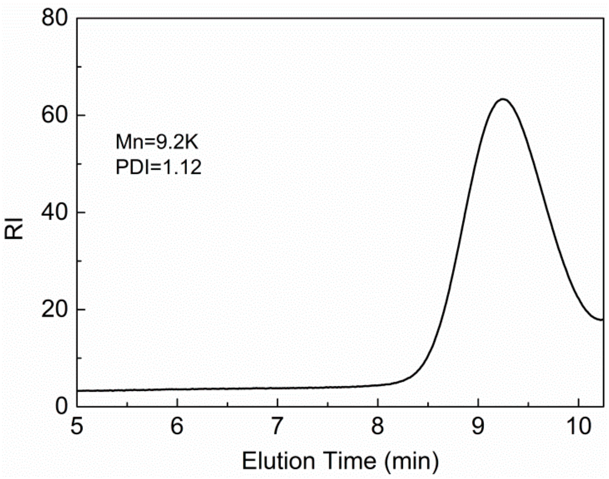


**Figure S5.** GPC trace of P(CPMI-alt-St).

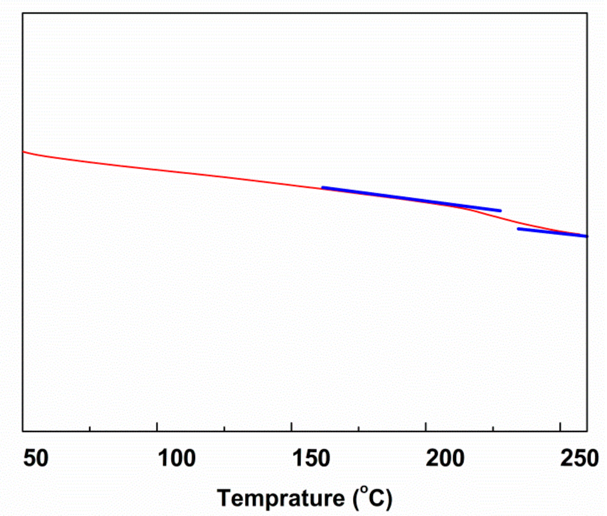


**Figure S6.** DSC trace of P(CPMI-alt-St).

© 2018 by the authors. Submitted for possible open access publication under the terms and conditions of the Creative Commons Attribution (CC BY) license (http://creativecommons.org/licenses/by/4.0/).
